# Interaction between Oxytocin Genotypes and Early Experience Predicts Quality of Mothering and Postpartum Mood

**DOI:** 10.1371/journal.pone.0061443

**Published:** 2013-04-18

**Authors:** Viara Mileva-Seitz, Meir Steiner, Leslie Atkinson, Michael J. Meaney, Robert Levitan, James L. Kennedy, Marla B. Sokolowski, Alison S. Fleming

**Affiliations:** 1 Institute of Medical Science, University of Toronto, Toronto, Ontario, Canada; 2 Department of Psychology, University of Toronto Mississauga, Mississauga, Ontario, Canada; 3 Women’s Health Concerns Clinic, St. Joseph’s Healthcare, Hamilton, Ontario, Canada; 4 Department of Psychiatry & Behavioral Neurosciences, McMaster University, Hamilton, Ontario, Canada; 5 Department of Psychology, Ryerson University, Toronto, Ontario, Canada; 6 Sackler Program for Epigenetics and Psychobiology at McGill University and Douglas Mental Health University Institute, Montreal, Quebec, Canada; 7 Center for Addiction and Mental Health, Toronto, Ontario, Canada; 8 Deparment of Psychiatry, University of Toronto, Toronto, Ontario, Canada; 9 Department of Ecology and Evolutionary Biology, University of Toronto, Toronto, Ontario, Canada; 10 Fraser Mustard Institute for Human Development, University of Toronto, Toronto, Ontario, Canada; Wayne State University, United States of America

## Abstract

Individual differences in maternal behavior are affected by both early life experiences and oxytocin, but little is known about genetic variation in oxytocin genes and its effects on mothering. We examined two polymorphisms in the oxytocin peptide gene *OXT* (rs2740210 and rs4813627) and one polymorphism in the oxytocin receptor gene *OXTR* (rs237885) in 187 Caucasian mothers at six months postpartum. For *OXT,* both rs2740210 and rs4813627 significantly associated with maternal vocalizing to the infant. These polymorphisms also interacted with the quality of care mothers experienced in early life, to predict variation in maternal instrumental care and postpartum depression. However, postpartum depression did not mediate the gene-environment effects of the *OXT* SNPs on instrumental care. In contrast, the *OXTR* SNP *r*s237885 did not associate with maternal behavior, but it did associate with pre-natal (but not post-natal) depression score. The findings illustrate the importance of variation in oxytocin genes, both alone and in interaction with early environment, as predictors of individual differences in human mothering. Furthermore, depression does not appear to have a causal role on the variation we report in instrumental care. This suggests that variation in instrumental care varies in association with a gene-early environment effect regardless of current depressive symptomatology. Finally, our findings highlight the importance of examining multiple dimensions of human maternal behavior in studies of genetic associations.

## Introduction

Early life experiences including adversity and stress influence development in many mammals. Early social isolation in rats results in a high proportion of mothers who are erratic, less attentive, and show reduced licking and grooming [1], [2]. Maternally deprived monkeys are more likely to reject nursing attempts by their own infants, they are less likely to ‘retrieve’ a crying infant, and tend to engage in more physical aggression towards their infants [3]. Human mothers who have experienced early adversity also show problematic mothering. They are more likely to be abusive and neglectful or to be less sensitive and responsive to their babies [4]. In all of these species, including humans, negative parenting experiences are transmitted across generations [5]–[7]. However, not all mothers are affected to the same extent. Differences in vulnerability to early experiences are related to protective factors throughout life, including nonparent support in early development, social support networks, and marrying a supportive spouse [8], [9]. Differences in vulnerability may also be influenced by individual differences in genetic profile [10]. Yet the role of genetic variation is largely unexplored in the study of individual differences in mothering.

In the present study, we first explored associations between genetic variation on both the oxytocin peptide-coding gene (*OXT*) and the oxytocin receptor gene (*OXTR*), and variations in human maternal care. The oxytocin system is an excellent candidate gene system in the maternal context. Extensive pharmacological, physiological, and behavioral evidence points to a role of oxytocin in mammalian mothering [11]–[15]. Oxytocin promotes maternal behavior in rats and sheep [16], [17] and oxytocin receptor antagonists inhibit the onset of rat maternal behavior [18]. Oxytocin receptor levels correlate with natural variations in maternal licking and grooming in rats [19] and with maternal behavior toward foreign lambs in sheep [20]. In humans, oxytocin is clearly associated with aspects of mother-infant relationships [21]. Plasma oxytocin levels in pregnancy predict attachment to the foetus [22], whereas postpartum serum and salivary oxytocin levels predict maternal behaviors toward infants [23], [24]. To date, two studies show associations between *OXTR* genotype and observed maternal sensitivity [25] and touching of the infant [26]. In contrast, genetic variants in the *OXT* gene are unexplored in association with human mothering.

Next, we examined the moderating effects of *OXT* and *OXTR* genotypes on the relationships between mothers’ early life experiences and present maternal care. Early experiences correlate with subsequent functioning of the oxytocin system in many mammalian species. Greater maternal licking/grooming in female rat pups predicts greater oxytocin receptor binding in adulthood [19], [27]. Early handling in female prairie voles predicts decreased oxytocin receptor binding in the nucleus accumbens and bed nucleus of the stria terminalis, two regions implicated in the regulation of maternal behavior [28]. Early maternal separation in both rhesus macaques and humans predicts lower adult levels of OXT in the cerebrospinal fluid [29], [30]. Moreover, the influences of early experience on oxytocin function may affect later social [28] or maternal behavior [31]. Therefore, genetic variation in the *OXT* and/or *OXTR* genes may moderate the extent to which early life experiences influences oxytocinergic function and associated behavior.

Finally, we explored the possibility that the interactive effect of *OXT* genotype and early experiences on behavior will be mediated by postpartum maternal mood state. Mothers with postpartum depression are more intrusive and irritable, and respond with less attentiveness and sensitivity to infants [32]–[38]. Depression itself is associated with early adversity and genetic variation [39]. A history of childhood adversity, including parental factors such as abuse, insecure attachment, rejection and lack of warmth [40], is a major risk factor for depression. Among the many factors that moderate and/or mediate the relationship between early adversity and adult depression are the timing of the early adversity [41], the presence of social support during development [42], oxytocin function [43] and *OXTR* genotypes [44]. Moreover, *OXTR* genotype is directly related to depression [39].

We explored these three questions in a sample of N = 187 Caucasian mothers during interactions with their six-month old infants. Early life experiences were assessed through questionnaires about abuse, neglect, childhood relationships with a mother’s own parents, and consistency of caregivers across childhood. There were four outcome measures of observed maternal care: orienting away from the infant, infant-directed vocalizing, instrumental care, and overall sensitivity of maternal responses. Firstly, the extent of maternal orienting away from the infant may be a measure of maternal distractibility. Orienting away is associated with both serotonin transporter and dopamine D1 receptor genotypes and is negatively correlated with maternal sensitivity [45], [46]. Secondly, maternal sensitivity, or the overall rating of maternal quality of interactions with the infant, associates with genetic variation in the serotonin transporter, *OXTR* [25], [26], and the vasopressin receptor gene [47], but not in the dopamine D1 or D2 receptor genes [45], [46]. Thirdly, maternal vocalizations to the infant, relate both to genetic variation in the dopamine D2 receptor and to maternal depression [33], [46]. The previous findings indicate that the maternal phenotypic outcomes we use in the present analysis have shown sensitivity in prior genetic association studies, and also highlight the potential specificity of polymorphisms relating to different dimensions of mothering phenotypes. Finally, instrumental care includes adjusting the infant’s clothing and grooming her face, nose, ears, etc. Maternal grooming is present across many mammalian species and has been related to oxytocin signalling in rats [19], [48] and monkeys [49]. However, in humans, younger and less experienced mothers exhibit more instrumental care (and fewer affectionate behaviors like kissing and caressing) [50]. Furthermore, employed mothers spend more time socially engaging with their infants than performing instrumental care [51]. This suggests that the appropriateness of instrumental care in humans may context specific. The previous studies highlight the need to investigate multiple dimensions of human maternal behavior.

## Methods

### Participants

Subjects were part of the longitudinal study on Maternal Adversity, Vulnerability, and Neurodevelopment (MAVAN). The study follows two cohorts of mothers and their infants: one in Montreal, QC, and another in Hamilton, ON, Canada. Behavioral data were available for the Hamilton cohort of mothers; the present study examines this cohort. Ethnic descent in this sample was mostly Caucasian (90%), with 3% mixed ethnicity, 2% African, 1.5% Hispanic, and 1% East Indian; the remainder were unspecified. This ethnic distribution is typical of the greater Hamilton region. Allele frequency distributions can differ across ethnic groups [52] and heterogeneous ancestry samples can reduce power [53]. Thus we examined the 187 Caucasian mothers in the Hamilton cohort. Subjects were ages 18–45 and recruited in their second trimester of pregnancy (weeks 12 to 24) from referrals to the St. Joseph’s Health Center (SJHC) Women’s Health Concerns Clinic and SJHC Ultrasound Department, Hamilton, Ontario, Canada. Two hundred and fifty-five mothers were originally enrolled. Fifty-one subjects were excluded due to: attrition (n = 28), premature delivery (n = 18), stillbirth or termination (n = 3), or involvement of the Children’s Aid Society (n = 2). A Chi-square test with Yates’ continuity correction revealed that the mothers who were lost to attrition did not differ significantly from the mothers who participated in the study on the level of intake adversity (including past or present psychiatric disturbances, early life abuse or neglect, or significant stressful life events) (χ^2^ (1, N = 249) = 0.57, p = 0.45). Most subjects reported having a partner (94%). Mean (±SD) maternal age was 31.2 (±4.9); mean maternal education was 4.81 (±2.3) on a scale of zero to ten, where zero is ‘not completed high-school’, and ten is ‘post-graduate degree’.

### Procedure

Subjects signed written consent to participate in the MAVAN project. Ethics approval for this study was obtained from the ethics review boards at the University of Toronto, Toronto, ON; and St Joseph’s Healthcare, Hamilton, ON. We assessed mothers and their children through questionnaires, diagnostic tools, and behavioral tasks, during 20 home and lab visits starting at weeks 12 to 24 of pregnancy and continuing until 72 months postpartum. Participants received $25 compensation after each visit.

#### Video-recorded mother-infant interaction

We assessed maternal behavior and maternal sensitivity by video-coding 30-minute in-home mother-infant recordings at six months postpartum. During the first 20 minutes mothers were free to interact with their infants as normal, but without feeding or changing the infants. In the last ten minutes mothers completed self-report questionnaires in the presence of their infants, to assess responsiveness during maternal divided attention.

One hundred and fifty-eight of the 187 Caucasian-sample mothers agreed to be recorded. These mothers did not differ significantly from mothers who did not participate in the video recording with respect to having prior children (parity), prenatal household income, maternal age, or maternal education.

#### Maternal and infant behavior

The first 20-minutes of the mother-infant interactions were coded by two raters using the Behavioral Evaluation Strategies and Taxonomies (BEST) coding system (Educational Consulting, Inc. Florida, US) [50]. This analysis generated duration and frequency data for multiple maternal behaviors by use of a computer keyboard with keys indexed for each behavior. We limited the analyses to behaviors that were present in 15% or more of the mothers. Inter-rater reliability was high (r = 0.80, n = 10). We quantified the following maternal behaviors as major outcomes: *orienting away from infant* (frequency), *infant-directed vocalizing* (duration), and *instrumental care* (duration). *Orienting away frequency* was the number of times a mother’s gaze was directed away from the infant’s head or body. This measure was transformed with the natural log (Ln) transformation due to positive skew. *Infant-directed vocalizing* was defined as any infant directed speech including motherese, nonsense words, and onomatopoeic sounds. All mothers vocalized during the 20-minute recordings and the measure had a normal distribution. *Instrumental care* included all maternal grooming and cleaning of the infant’s body and face, as well as adjusting clothes, bibs, soothers, and hats. We corrected the skew in this measure by using the square root transformation. We quantified the following three infant behavioral durations: *reaching toward mother*, *smiling at mother*, and *crying.* These three durations were aggregated to obtain a total duration of *infant activity*. We later used this measure as a covariate in the regression analyses.

#### Maternal sensitivity

A single rater coded the full-length 30-minute videos for maternal *sensitivity* using the Ainsworth Maternal Sensitivity Scales [54] (n = 158), which contain four subscales: Cooperation, Accessibility, Acceptance, and Sensitivity. Mothers receive a rating (1–9) on each of the four subscales, and these are later aggregated to obtain a total Ainsworth Sensitivity Score. Inter-rater reliability was high across the individual subscales (r = 0.83 across all four subscales; n = 10) in comparison with another experienced rater. Raters were blind to subjects’ genotype and early experience status.

#### Maternal mood

We used the Center for Epidemiological Studies Depression Scale (CES-D; [55]), a 20-item self-assessment tool, to assess maternal mood at 12–24 weeks of pregnancy and again at 6 months postpartum. Each item contains response options scored between 0 and 3, for an overall score ranging from 0 (no depression) to 60 (highest level of depression). The CES-D scale is one of the most widely used depression scales [56] with excellent psychometric properties across clinical and community samples, English speaking and non-English speaking [57], [58].

#### Early care quality

Early experience was assessed from the following three questionnaires. The Life History Calendar (LHC; [59]) assessed the number of experienced changes in primary caregiver during the subjects' first 18 years of life. The Childhood Trauma Questionnaire (CTQ; [60]), assessed five types of childhood trauma: physical, emotional and sexual abuse; emotional and physical neglect. The Parental Bonding Instrument (PBI; [61]), assessed quality of parenting experienced during the subjects' first 16 years of life. The PBI and CTQ have good psychometric properties [62], [63]. The LHC and CTQ were delivered at 12–24 weeks of pregnancy, whereas the PBI was delivered at 6 months postpartum. The PBI has been shown to exhibit stability over time [63], [64]. We used factor analysis to reduce the multiple dimensions within these three questionnaires to obtain an *early care quality* factor [45]. The *early care quality* values range from negative (high early life adversity) to positive (low early life adversity). This dimension was the first principal component (of two) derived from principal component analysis (quartimax rotation) with nine input variables from CTQ, PBI, and LHC, and had an eigenvalue of 4.0. Early experience dimensions that loaded highly on care quality were the following: CTQ physical abuse, CTQ emotional abuse, CTQ physical neglect, CTQ emotional neglect, PBI maternal care and PBI maternal overprotection (intercorrelations between these six dimensions were significant and the absolute value Kendall’s tau coefficients ranged between 0.25 and 0.56, p<0.01 across all cases). Missing values in the derived early care quality dimension were imputed with stochastic regression single imputation in SPSS, where the original nine CTQ, PBI and LHC variables were used as predictors (methods described in [45]).

#### Buccal cell swabs and genotyping

We collected subjects’ DNA through buccal swabs. DNA extraction and genotyping were carried out at the Center for Addiction and Mental Health (CAMH), Toronto, Ontario, Canada. Because of the relative lack of known functional polymorphisms in the two candidate genes, particularly the *OXT* peptide gene, we chose the SNPs based on available evidence of associations with mood disorders [65] and antipsychotic treatment response [66]. Furthermore, there is evidence that the *OXTR* SNP in this study (rs237885) is part of a haplotype block for *OXTR*, which is significantly associated with pro-social behaviors [67]. Rs2740210 is in the 3′ flanking region of the *OXT* gene, rs4813627 is 56 kb downstream of the *OXT* gene, and rs237885 is in the intron region of the *OXTR* gene. The genotypes of the *OXT* polymorphisms were determined by the Taqman assay method using the ABI PRISM 7000 (Applied Biosystems, Foster City, California, USA). The IDs for on-demand assays available from ABI were as follows: C__16061225_10 (rs2740210), C___2712196_10 (rs4813627), and C___3290319_1_ (rs237885).

### Analysis

Hardy-Weinberg Equilibrium tests and linkage disequilibrium tests were carried out with the *genetics* package in the open-source statistical framework R (http://cran.r-project.org/). We first tested for main effects of maternal *OXT* genotype on maternal behavioral outcomes using analysis of variance (ANOVA). *OXT* SNPs (rs2740210 and rs4813627) were entered in the models both separately and together. The direction of effects was the same, but because of high LD (see above) results are presented for the analyses in which these SNPs are entered together.

Next, we used multivariate regression models to test for genotype×early experience (G×E) effects on 1) maternal behavior and 2) maternal mood (postnatal depression, CES-D score). We first ran full models with genotype, early experience, and their cross-product as predictor variables; maternal age, education, parity (whether this was the mothers’ first child), and postnatal depression score (in the model predicting maternal behavior) as maternal covariates; and infant gender and activity as infant covariates. Model reduction with the F-test enabled the removal of non-significant covariates for the “reduced models”. We present output from both the full and the reduced models in the regression tables.

For the SNP main effect models and the G×E regression models, we used multiple imputation to impute values for missing data in the behavioral outcomes (*orienting away from infant, infant-directed vocalizing, instrumental care, maternal sensitivity, and infant activity*), with the *Hmisc* package in R. Imputations were performed with predictive mean matching and Bayesian approximation methods over ten iterations, and results were combined with the fit.mult.impute function, which returns likelihood ratio test chi-squared values as a test of overall fit of the test model compared to the null model (intercept only). This function also returns average beta coefficients. Imputations were performed for the following percentages of missing data in the sample (N = 187): 14% for behavioral outcomes, including maternal and infant behaviors; 9% for parity; 14% for maternal education; and 12% for CES-D Depression scores.

For mediation analyses, we followed steps outlined in Edwards and Lambert [68] to test a full mediation moderation model. We tested a full moderated mediation model [68] by entering our predictors, moderators, and mediators into the following regression equations:
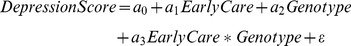
(1)

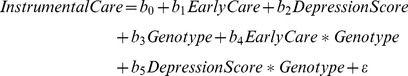
(2)


Where *Genotype* is *OXT* rs2740210 (coded as C/C = 0; and A/C or A/A = 1) and *Depression Score* is maternal CES-D score at 6 months, centered at the standard cut-off score of 16. We used this model to derive path coefficients for a direct effect of early experience (*early care quality*) on *instrumental care*, Y; and indirect paths (A) from *early care quality* to depression (CES-D) and (B) from CES-D to *instrumental care*. Each of the three paths was modeled to be potentially moderated by genotype. Regression coefficients and bias-corrected boot-strapped confidence intervals (n = 1000) were derived using the boot and boot.ci functions in the *boot* package in R. For mediation analyses we used the original rather than the imputed data.

Finally, path models using prenatal depression as a mediator were also examined, to strengthen any arguments based on the previous model where the mediator (depression) and parental outcome (instrumental care) occur at the same time-point (6 months postpartum). We present the model with prenatal depression in the supplementary materials.

## Results

Sample characteristics and study variables are described in Table S1. The three SNPs were in Hardy-Weinberg equilibrium (Table 1). There was significant linkage disequilibrium (LD) between the *OXT* SNPs rs2740210 and rs4813627 (D’ = 0.79, χ^2^ = 86.1, p<0.001). When SNPs are in high LD, the individual effects of SNPs in regression models should be interpreted cautiously. Results from haplotype analyses are presented in Table S2.

**Table 1:** Minor allele and genotype frequencies in the three *OXT/OXTR* SNPs.

	% Genotyped	Minor allele frequency	Genotype frequency		
OXT rs4813627	87	A (.50)	G/G (44)	A/G (73)	A/A (45)
OXT rs2740210	84	A (.31)	C/C (77)	A/C (63)	A/A (18)
OXTR rs237885	84	G (.38)	T/T (43)	G/T (72)	G/G (42)

There was a significant negative correlation between *infant-directed vocalizing* and frequency of *orienting away from the infant* (Spearman’s rho (158) = −0.3, p<0.01), and between frequency of *orienting away* and *sensitivity* (Spearman’s rho (158) = −0.19, p = 0.02) (Table 2). *Instrumental care* correlated negatively with maternal education (Spearman’s rho (139) = −0.17, p = 0.04), and positively with *infant activity* (Spearman’s rho (158) = 0.22, p<0.01). There was a trend for a positive correlation between instrumental care and *CES-D postnatal depression score* (Spearman’s rho (145) = 0.16, p = 0.05). Depression score negatively correlated with maternal education (Spearman’s rho (143) = −0.25, p<0.01) (Table 2). ANOVAs revealed no association between genotype and early maternal care quality (p<0.05 for all three SNPs tested).

**Table 2:** Correlations between maternal socioeconomic, behavioral, and mood variables.

	Orienting Away	Vocalizing	Instrumental Care	Ainsworth Sensitivity	Depression Score (CES-D postnatal)
Orienting away	**–**				
Infant-directed Vocalizing	**−.30** ***	–			
Instrumental Care	.03	.06	–		
Ainsworth Sensitivity	**−.19** *	.13	−.11	–	
Maternal Age	.06	−.10	−.10	.09	−.15∼
Education	.05	−.15	**−.17** *	.14	−.25**
Parity	.14	.00	−.05	−.12	.12
Depression Score (CES-D prenatal)	−.07	.01	.11	−.10	**.60** ***
Depression Score (CES-D postnatal)	−.01	.02	.16∼	−.14	–
Infant Gender	−.07	−.01	−.02	−.06	.06
Infant Activity	**−.19** *	.13	.22**	−.15∼	.09

NOTE: Values are Spearman’s rho coefficients, 2-tailed; ∼ p<0.1;

* p<0.05;

*** p<0.001; N = 128–187.

### Main Effects of Genotype

**Figure 1:**
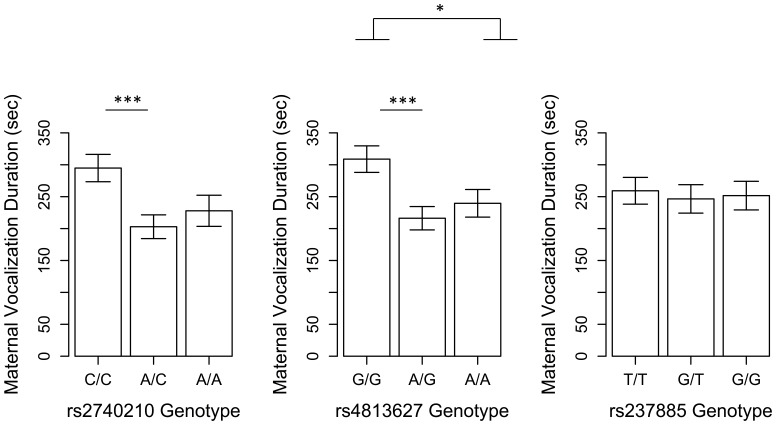
Main effects of OXT genotype on infant-directed vocalizing. Mothers with C/C and G/G genotypes for rs2740210 and rs4813627, respectively, vocalize significantly longer to their infants; values plotted represent non-imputed values of the original sample; regression coefficients and statistics presented here and in the text are based on multiply imputed values for maternal outcomes (see Methods); ***p<0.001; †p<0.1; values are means ± SEM.

There were significant main effects of both rs2740210 A/C and rs4813627 A/G genotype (reference group was rs2740210 C/C and rs4813627 G/G respectively) on *infant directed vocalizing* (β = −52.8, t(147) = −2.09, p = 0.04 and β = −56.3, t(147) = −2.06, p = 0.04, respectively). Together rs2740210 and rs4813627 genotypes explained a significant proportion of variance in *infant-directed vocalizing* (R^2^ = 0.10, LR χ^2^ (4, N = 152) = 15.7, p = 0.004) (Fig. 1). Based on the pattern of effects in Figure 1, we grouped genotypes A/C and A/A for rs2740210 and genotypes A/G and A/A for rs4813627. For rs2740210 the A/A genotype group had quite a small sample size (N = 18), providing further justification for this grouping. Haplotype analyses (Table S4) revealed a consistent pattern of results.

### Genotype, Experience, and Mothering

**Figure 2:**
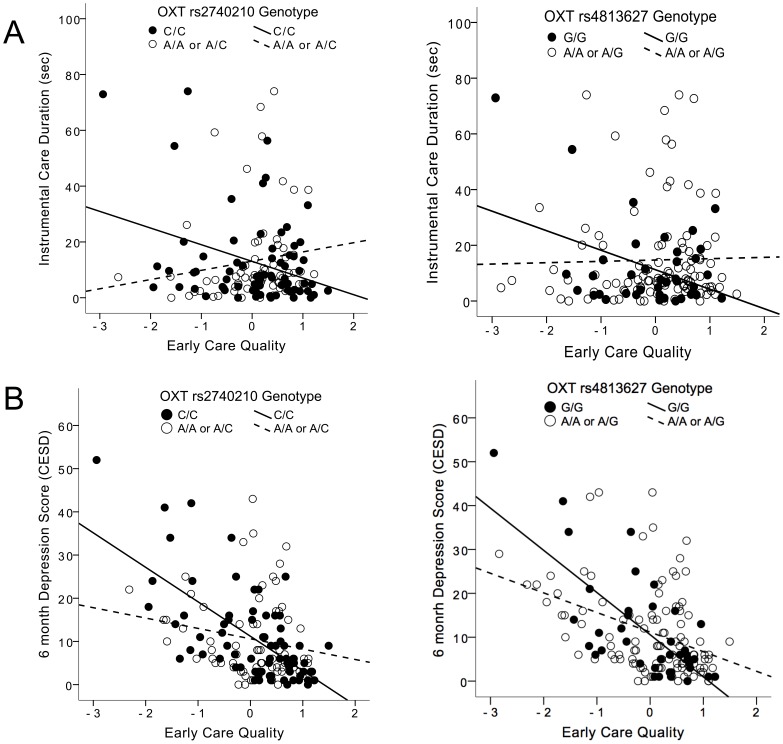
Interaction between *OXT* genotypes (rs2740210 and rs4813627) and maternal self-reported early care quality (factor derived from multiple early experience measures, see Methods) on (A) duration of instrumental care during a 20 minute maternal-infant recorded interaction and (B) depression score assessed with the CES-D depressioni scale at 6 months postpartum. Figures do not take into account covariates used in analyses (Tables 3 and 4).

In both the full and reduced models for rs2740210 and rs4813627 there were significant main effects of *early care quality* on the total duration of maternal *instrumental care* (Table 3). Moreover, there was a significant *genotype*×*early care quality* on the overall duration of maternal *instrumental care* (Fig. 2, Table 3). For rs2740210 genotype C/C and rs4813627 genotype G/G, respectively, mothers engaged in less instrumental care as reported *early care quality* increased. For the other genotype groups, this relationship was reversed: mothers had longer total duration of *instrumental care* with increasing *early care quality.* Finally, there was a main effect in these models of *infant activity,* but no other infant or maternal covariate tested.

**Table 3:** Beta regression coefficients (*t*-statistics in brackets) for analyses predicting maternal instrumental care duration.

	OXT rs2740210 (N = 158)			OXT rs4813627 (N = 162)		
	Full Model	Reduced Model		Full Model		Reduced Model
Intercept	11.57 (0.98)		2.54 (0.50)	7.04 (0.60)	0.13 (0.02)	
Genotype (G)	0.99 (0.35)		0.97 (0.34)	2.72 (0.82)	3.16 (0.96)	
Early Care Quality (E)	−4.69 (−1.68)∼		−5.67 (−2.50)*	−5.67 (−1.60)	−6.61 (−2.13)*	
Maternal Age	−0.13 (−0.39)		−	−0.07 (−0.22)	−	
Parity	−3.17 (−1.04)		−	−2.64 (−0.84)	−	
Education	−0.83 (−1.08)		−	−0.91 (−1.14)	−	
Postnatal depression score (CES-D)	0.14 (0.71)		−	0.10 (0.54)	−	
Infant Gender	−0.62 (−0.20)		−	1.10 (0.36)	−	
Infant Activity	2.68 (2.23)*		2.84 (2.41)*	3.09 (2.56)*	3.11 (2.62)**	
G×E	8.37 (2.15)*		8.60 (2.64)**	7.49 (1.96)*	7.91 (2.23)*	
R^2^ (adj.)	0.10		0.08	0.08	0.06	
Likelihood Ratio χ^2^	26.79**		18.02**	22.01**	14.70**	
df	(9, 147)		(4, 153)	(9, 151)	(4, 157)	

NOTE: For Tables 3 and 4, coefficients represent averages from ten imputations; the ‘full model’ contains several maternal and infant covariates whereas reduced model (obtained after F-test reduction in number of predictor variables) contains only variables that contribute significantly to the model. ∼p<0.10,

* p<0.05,

** p<0.01.

There were no main effects of early care quality or interactions of care quality with *OXT* genotypes in relation to *maternal sensitivity* or *orienting away*. Because of high linkage disequilibrium between the two *OXT* SNPs, the results of the regressions using both SNPs in the model should be interpreted cautiously. There were no effects of *OXTR* genotype (rs237885) on any maternal measure.

### Genotype, Experience, and Depression

There were significant *genotype*×*early care quality* interactive effects (for both rs2740210 and rs4813627) on maternal depression score (CES-D) at 6 months (Fig. 2, Table 4). Mothers scored lower on depression as the *early care quality* increased, but the slopes for genotypes C/C (rs2740210) and G/G (rs4813627) were more negative, indicating that early life experience had a stronger moderating effect in this group of mothers than in mothers with other genotypes (A/A or A/C for rs2740210; and A/A or A/G for rs4813627) (Table 4). For rs4813627, there was also a significant main effect of maternal education, so that depression score decreased with increasing education. There were no effects of *OXTR* genotype (rs237885) on depression levels at 6 months postpartum, but when we used a prenatal depression measure, there were significant associations between rs237885 genotype G/G, which explained a significant proportion of the variance (R^2^ = 0.07, LR χ^2^ (2, N = 154) = 11.72, p = 0.003).

**Table 4:** Regression analyses predicting maternal depression score (CES-D) at 6 months postpartum.

	OXT rs2740210 (N = 158)			OXT rs4813627 (N = 162)	
	Full Model		Reduced Model	Full Model	Reduced Model
Intercept	20.85 (2.81)**	11.23 (9.57)***		16.45 (2.19)*	12.99 (3.52)***
Genotype (G)	−0.34 (−0.19)	−0.34 (−0.18)		0.66 (0.37)	0.63 (0.36)
Early Care Quality (E)	−7.45 (−5.92)	−7.77 (−6.28)***		−9.35 (−5.58)***	−9.36 (−5.55)***
Maternal Age	−0.29 (−1.31)	−		−0.20 (−0.89)	−
Parity	0.98 (0.52)	−		1.65 (0.92)	−
Education	−0.37 (−0.97)	−		−0.70 (−1.82)∼	−0.86 (−2.54)*
Infant Gender	1.30 (0.80)	−		1.77 (1.09)	−
Infant Activity	0.09 (0.12)	–		0.55 (0.76)	–
G×E	5.33 (2.56)*	5.89 (2.91)**		5.83 (2.97)**	5.95 (2.98)**
R^2^ (adj.)	0.23	0.21		0.26	0.25
F	48.98***	39.52***		56.82***	51.05***
Df	(8, 148)	(3, 154)		(8, 152)	(5, 156)

NOTE: Table coefficients represent averages from ten imputations. ∼ p<0.10,

* p<0.05,

** p<0.01,

*** p<0.001.

### Mediation/Moderation Model

**Figure 3:**
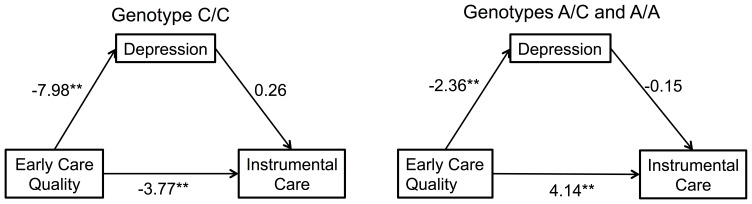
Path models depicting relationships between early experience, postnatal depression, and maternal instrumental care in two genotypes of rs2740210: C/C and A/C+A/A using coefficients from **Table 6**. Significance values are based on bias-corrected bootstrap adjusted confidence intervals. Postnatal depression is not a significant mediator of the early care quality associations with instrumental care, for either genotype.

In mediated moderation analysis, we compared rs2740210 genotypes C/C with genotypes A/C and A/A, and found direct effects and indirect effects in the model from early experience to instrumental care. For both rs2740210 genotypes, there was a significant negative association between early care quality and postnatal depression (path coefficients are −7.98 (C/C) and −2.40 (A/C; A/A)) (Fig. 3; Table 5 and Table 6). The direct path from early care quality to instrumental care was significant and negative for C/C mothers (−3.77); and significant and positive for A/C or A/A mothers (4.30). However, the path from depression to instrumental care was not significant for either C/C or A/C and A/A genotypes of rs2740210.

**Table 5:** Coefficient estimates from path models.

	Equation 1^a^				Equation 2 b					
	Early Care	Genotype^c^	Early Care*Genotype	R^2^	Early Care	Depression^d^	Genotype	Early Care*Genotype	Depression*Genotype	R^2^
Coefficients	−7.98**	−0.72	5.62**	0.24***	−3.77*	0.26	−0.26	7.91**	−0.11	0.04∼
CI 2.5%	−10.10	−3.91	6.40		−11.22	−0.20	−10.05	6.65	−1.11	
CI 97.5%	−7.81	1.59	6.45		−0.59	1.14	7.00	13.56	0.54	

NOTE: ^a^coefficients were derived using Equation 1 (Methods);

b Coefficients were derived using Equation 2 (Methods);

c OXTrs2740210 genotype (grouped as genotype C/C versus genotype A/C and A/A);

d Depression measured postnatally, with the CES-D scale. CI: bias-corrected confidence intervals which are Bootstrap adjusted values;∼p<0.1,

* p<0.05,

** p<0.01.

**Table 6:** Simple effects of path models (using postnatal depression score as a mediator).

	Stage		Effect		
rs2740210 Genotype	First	Second	Direct	Indirect	Total
C/C	−7.98**	0.26	−3.77**	−2.06*	−5.85**
A/C and A/A	−2.36**	−0.15	4.14**	−0.35	3.79**
Differences	5.62**	−0.11	7.91**	1.72	9.63**

Note: N = 129–140. Simple effects computed using equation 25 in Edwards and Lambert (2007) using coefficients estimates from Table 5. Zs = 0 for C/C and 1 for A/C and A/A genotypes (rs2740210). Differences in simple effects were calculated by subtracting the A/C+A/A effects from the C/C simple effects. Significance values are based on bias-corrected boot-strap adjusted confidence intervals.

* p<0.05,

** p<0.01.

Models using prenatal depression revealed a similar pattern of effects for the C/C genotype mothers (significant first stage effect (early care depression) and direct effect (early care instrumental care)), and a similar direction (though not significant) of effects for the alternate genotype (Tables S3, S4, and Fig. S1). The differences between the effects in the two genotypes were significant. Thus the overall prenatal depression model had the same pattern of effects as the postnatal model.

## Discussion

There is accumulating evidence for the role of oxytocin in the regulation of human mothering [21]. We examined polymorphic variation on two oxytocin genes – one which codes for the oxytocin peptide (*OXT*), and one which codes for the oxytocin receptor (*OXTR*) – and found that polymorphisms in *OXT* (but not *OXTR*) associate with both infant-directed vocalizing and maternal instrumental care.

There was a main effect of genotype on vocalizing for both *OXT* SNPs (rs2740210 and rs4813627). Vocalizing to infants is a universal behavior of mothers that includes infant-directed speech, or “motherese”, characterised by exaggerated prosodic cues and is present in all studied languages [69]. The distinctive vocal characteristics of infant-directed speech are believed to act as cues for the infant, and to aid communication and language learning and promote closeness [70], [71]. Maternal speech can be further subdivided, for instance into affect-salient and information-salient content [70]. In the present research, we opted for quantifying the entire duration of the vocalization. In this sense, though a large component of these vocalizations are “motherese”, our measure of infant-directed vocalization is a measure of maternal engagement with the infant.

We previously showed that genetic variation in the dopamine D1 and D2 receptor genes (*DRD1* and *DRD2*) associates with individual variation in human maternal care, including infant-directed vocalizing [46]. Specifically, there was a notable association between *DRD2* genotypes and haplotypes and maternal infant-directed vocalizing. The link between genetic variation in both the *OXT* and *DRD2* genes and individual differences in the duration of maternal vocalizing to infants is plausible. Oxytocin neurons express DRD2-like receptors in the rat brain [72]. There is also evidence for a functional link between dopamine and oxytocin in the rat mesolimbic dopamine pathway, which is important not only for reward-processing but also for the expression of maternal behavior [73]. Oxytocin infusion into the ventral tegmental area (VTA) enhances the dopamine signal in the nucleus accumbens [74]. Moreover, antagonist oxytocin infusion into the VTA disrupts rat maternal care [18]. Therefore genetic variants on these genes may have similar or even interactive effects on behavioral outcomes. Our sample size precluded us from examining gene-gene interactions between oxytocin and dopamine pathway genes, but this should be an important future step for studies in mothering. The many complex neural pathways that regulate mothering do so by acting in concert; therefore multiple genes and genetic polymorphisms underlying these pathways are likely to influence mothering. Indeed, the prior evidence relating genotypic variation in the serotonin transporter gene [25], [45], the vasopressin peptide gene [47], the dopamine D1 and D2 receptor genes [46], [75], and the oxytocin receptor gene [25], to differences in mothering suggest opportunities for studying gene-gene interactions in individual variation in maternal care, given large enough samples.

In the present report we found that maternal *OXT* genotype also associates with maternal instrumental care, in interaction with early maternal life experience. Mothers with genotypes C/C or G/G (for rs2740210 and rs4813627, respectively) displayed shorter overall instrumental care duration with increasing early care quality; for mothers with the other two genotypes for these SNPs, instrumental care duration *increased* with increasing early care quality (Fig. 2A).

In non-human animals, grooming is an essential caregiving behavior. Maternal rat dams spend considerable amounts of time in the early postpartum licking/grooming their pups and variations in the time spent licking/grooming predicts a number of developmental and behavioral outcomes in the growing and adult offspring [27], [76], [77]. In humans, mothers groom as part of instrumental or caretaking behaviors. Younger mothers engage in more instrumental caretaking behaviors than older mothers [50]. Instrumental behavior did not correlate with maternal age in our sample, but negatively correlated with maternal education. The more educated the mother, the *less* time she spent performing instrumental tasks like grooming, adjusting, and cleaning the infant. Indeed, research supports the notion that maternal education and employment status influence how mothers allocate their time while interacting with their infants. For instance, employed women engage in significantly less instrumental care, and significantly more socialization with their infants than non-employed women [51]. However, instrumental care is positively correlated with maternal age and education in this study [51], whereas education was *negatively* related to instrumental care in our sample. These differences may arise from the inherent differences in instrumental care between the two studies. In our study, free-play interactions were designed to *minimize* instrumental care – mothers were instructed to refrain from changing diapers, breast- or bottle-feeding, bathing, or other time-consuming instrumental care behaviors. In contrast, in the Huston & Rosenkrantz Aronson study [51], mothers filled out questionnaire reports about the total duration of caretaking activities over a full two days.

There was also a notable main effect of infant activity on instrumental care: the longer the infants cried, smiled, or reached toward their mothers, the longer the duration of maternal instrumental care, though this relationship may be bi-directional (Table 3). In other words, infants may cry, reach, or smile more in response to maternal caretaking behaviors. Conversely, mothers may engage in instrumental behavior in response to infant fussiness or signals of attention-seeking. There is a potential role for infant genotype on this dyadic interaction, which should be studied in future. Observed instrumental behaviors during a free-play interaction therefore likely represent an important aspect of maternal strategizing; for some mothers instrumental care may be more prominent than for others, and we have shown that the levels of maternal instrumental care is predicted by the interactive effects of genotype and early life experiences.

Instrumental caregiving behaviors may also be part of a more generally intrusive (over-stimulating, controlling) style of mothering sometimes associated with depressed mothers [32]. Indeed, we found a trend for a negative correlation between depression score and instrumental care in our cohort of mothers (Table 2). We subsequently explored the possibility that depression mediates the genetic associations with mothering. Moreover, early stress influences oxytocin signalling in adulthood. We thus tested the interaction between *OXT* genotype and early care quality, and found a highly significant association on CES-D scores at 6 months postpartum (Table 4). However, when we tested these in a mediation/moderation model, depression (both prenatal and postnatal) showed no significant association with maternal instrumental care, regardless of maternal genotype. Although *OXT* genotype×early care quality predicted both maternal instrumental care and CES-D score, these appear to be separate effects rather than causally linked outcomes. There is support for this in the regression models, which show a significant main effect of infant activity on maternal instrumental care, but not on maternal postnatal depression.

In addition to being intrusive, depressed mothers are sometimes withdrawn - vocalizing less with their infants and looking away more often [78]. Yet there was no correlation between depression score and maternal infant-directed vocalization in our sample (Table 2). However, ours was not a clinically depressed sample and the majority of mothers scored below the clinical cut-off, 16. Moreover, mean CES-D score in the prenatal period was higher than in the postnatal period, possibly because mothers who presented with depression or depressive symptoms prenatally were seen by a psychiatrist and received behavioral or pharmacological therapy. It would be interesting to replicate this study in a clinically depressed sample of mothers. In such a sample, greater depressive symptoms may more directly influence mothering behavior, and may be a significant mediator of *OXT* genotype×early life experience influences on mothering.

Interestingly, the genotype groups that showed the longest duration of overall infant-directed vocalizing (Figure 1) also showed greater moderation by early environment on depression score. For rs2740210 and rs4813627, genotypes C/C and G/G, respectively, showed steeper negative slopes when CES-D scores were regressed onto early care quality (Figure 2). Variations in the quality of early care among these mothers had a greater effect on postpartum depression scores. Very similar findings are reported for the *OXTR* polymorphism rs2254298, which associates with depression and anxiety in adults [79] and, in the face of early adversity, associates with anxiety and depression in adolescent girls [44]. It is unknown if the two non-coding *OXT* SNPs in our study exhibit gene-gene interactions with the *OXTR* rs2254298 SNP in the above studies, or if they have similar or overlapping functions. There is an indication that rs2254298 influences amygdala volume, but this effect may be ethnicity-dependent as it is found in Asian, but not Caucasian populations [80], [81].

Another *OXTR* polymorphism, rs53576, has previously been shown to associate with maternal sensitivity [25] and depression [39]. We did not have available genotypes for this SNP, but did examine the rs237885 polymorphism, which also lies on the *OXTR* gene. We found a notable absence of associations between this SNP and any maternal outcome tested. The previously studied rs53576 is a silent (AG) mutation and along with the SNP we assessed, rs237885, lies in the intronic region of the *OXTR* gene, with no known effects on expression or protein function. Yet because rs53576 has effects in other association studies, future research should examine this SNP in association with early adversity for effects on maternal behavior.

None of the three SNPs we explored showed associations with maternal *sensitivity*, a global measure of maternal quality of care; or with *orienting away*, a measure of maternal responsiveness potentially related to attention. We have previously reported that *orienting away* associates with dopamine D1 receptor genotype and with genotype at the serotonin transporter-linked polymorphic region (5HTTLPR) [45], [46], and have argued that this measure reflects maternal inattention or distractibility. The lack of association between *OXT* SNPs and orienting away is not surprising. Oxytocin seems to have functions centered on sociality and affect, and therefore attention-related dimensions of mothering are unlikely to be influenced by genetic variation in the *OXT* gene.

One limitation to our study is the use of a retrospective measure of early care quality. However, the subcomponents of our measure were the Childhood Trauma Questionnaire (CTQ) and the Parental Bonding Instrument (PBI), both of which have long-term stability. The PBI has been examined in clinical samples where depressed individuals improved over time; in mothers who were followed from the early postpartum for 30 months [64]; and in a sample where scores were taken 20 years apart [63]. These studies indicate stability of scores across time, regardless of depression or postpartum status. Furthermore, there is a high correlation between CTQ (assessed prenatally) and the PBI (assessed at 6 months postpartum). Another limitation is the relatively small sample size for a genetics study and the lack of a replication sample. After exclusion of the non-Caucasian mothers, we had 187 remaining mothers. Quantification of complex phenotypes such as maternal behavior is time-intensive and this size of sample is quite large for psychological studies of this kind.

The present report is the first, to our knowledge, to examine genetic variation in the oxytocin peptide gene *OXT* in relation to maternal behavior. We examined two polymorphisms in the *OXT* gene (rs2740210 and rs4813627), and both showed a main effect on maternal vocalizing to the infant; as well as a significant interactive effect with early life experience on maternal instrumental care of the infant. The significant associations for type (i.e. vocalizing, instrumental care) but not quality (i.e. maternal sensitivity) of maternal care, highlights the importance of exploring multiple levels of behavioral phenotypes in genetic association analyses. Finally, it is important to keep in mind that mother-child interactions may change over time. Thus, they represent a dynamic relationship, which at different times may exhibit different influences from hereditary and non-hereditary factors. Genetic studies should begin to delve into the temporal stability of association between genetic variation and multiple outcomes over time.

## Supporting Information

Figure S1 Path models depicting relationships between early experience, prenatal depression, and maternal instrumental care in two genotypes of rs2740210: C/C and A/C using coefficients from Table 6. Significance values are based on bias-corrected bootstrap adjusted confidence intervals. Prenatal depression is not a significant mediator of the early care quality associations with instrumental care, for either genotype.
(TIFF)

Table S1 Sample characteristics and study variables.
(DOCX)

Table S2 OXT haplotypes and regression models with these haplotypes.
(DOCX)

Table S3 Coefficient estimates of path models (using prenatal CES-D).
(DOCX)

Table S4 Simple effects of path models (using prenatal prenatal depression score as a mediator).
(DOCX)
