# Supplementary figures and images for: Interaction between Oxytocin Genotypes and Early Experience Predicts Quality of Mothering and Postpartum Mood
Source: PLoS One. 2013 Apr 18;8(4):e61443. doi: 10.1371/journal.pone.0061443 (PMC3630168; doi:10.1371/journal.pone.0061443)

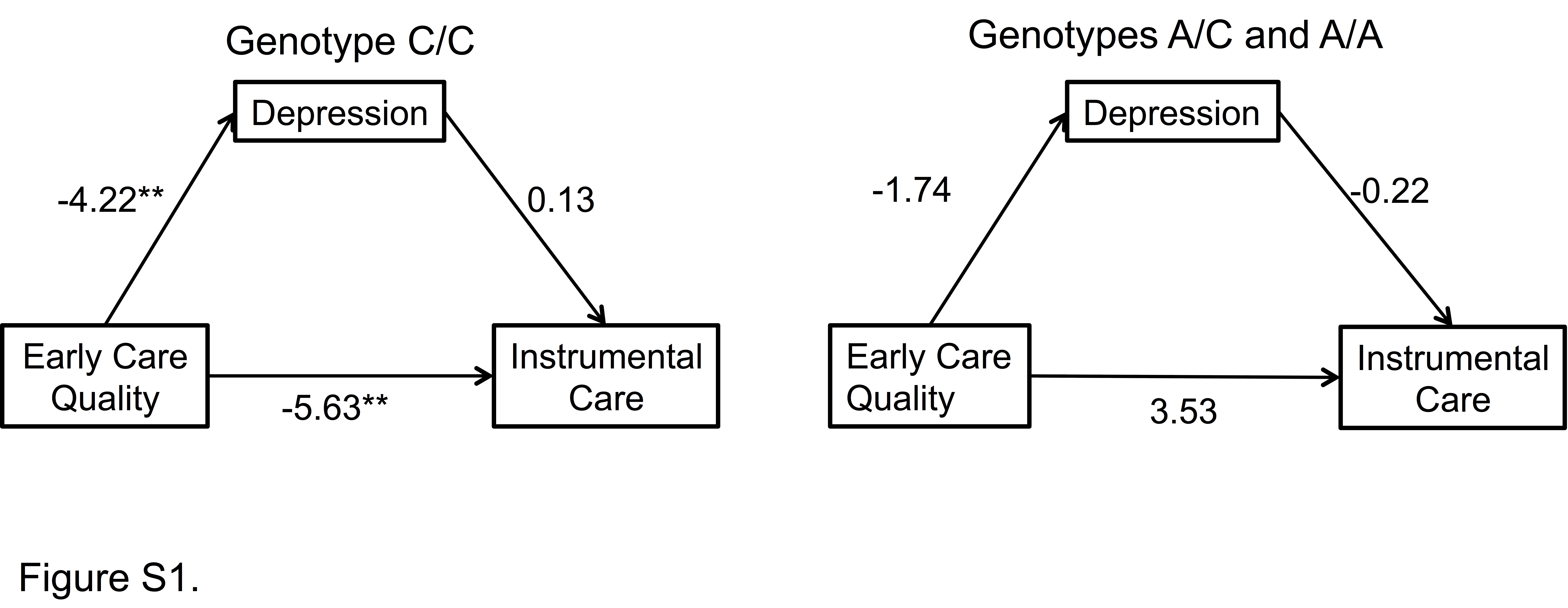

Supplement: Figure S1 — Path models depicting relationships between early experience, prenatal depression, and maternal instrumental care in two genotypes of rs2740210: C/C and A/C using coefficients from Table 6. Significance values are based on bias-corrected bootstrap adjusted confidence intervals. Prenatal depression is not a significant mediator of the early care quality associations with instrumental care, for either genotype. (TIFF) [file pone.0061443.s001.tiff]
