# Supplementary material for: Interaction between Oxytocin Genotypes and Early Experience Predicts Quality of Mothering and Postpartum Mood
Source: PLoS One. 2013 Apr 18;8(4):e61443. doi: 10.1371/journal.pone.0061443 (PMC3630168; doi:10.1371/journal.pone.0061443)
Supplement: Table S1 — Sample characteristics and study variables. (DOCX) [file pone.0061443.s002.docx]

Table S1. Sample characteristics and study variables

|  |  | N=158-187 |
| --- | --- | --- |
| Maternal Age |  | 31.2 (± 4.9) |
| Parity (first-time mothers) |  | 43.5% |
| Infant gender (male) |  | 61.0 % |
| Maternal Education |  | 4.8 (± 2.3) |
| Income |  | 14.0 (± 3.3) |
| Early Care Quality |  | 0.0 (± 0.9) |
| Depression score (CES-D), prenatal |  | 9.00 (± 17.0) |
| Depression score (CES-D), postnatal |  | 6.00 (± 12.0) |
|  |  |  |
| Vocalization (duration) |  | 242.2 (± 178.9) |
| Instrumental Care (duration) |  | 7.6 (± 13.9) |
| Sensitivity Average Score |  | 6.0 (± 1.3) |
| Orienting Away (Frequency) |  | 20.0 (± 18.5) |
| Infant Activity |  | 59.6 (± 77.6) |

Values are means (± St. Dev.) for normally distributed variables, medians (± IQR) for non-normally distributed variables, and percents for dichotomous variables.
