# Supplementary material for: Interaction between Oxytocin Genotypes and Early Experience Predicts Quality of Mothering and Postpartum Mood
Source: PLoS One. 2013 Apr 18;8(4):e61443. doi: 10.1371/journal.pone.0061443 (PMC3630168; doi:10.1371/journal.pone.0061443)
Supplement: Table S2 — OXT haplotypes and regression models with these haplotypes. (DOCX) [file pone.0061443.s003.docx]

Table S2. OXT haplotypes and regression models with these haplotypes

|  | OXT SNP | |  | Regression Models^b^ | | | |
| --- | --- | --- | --- | --- | --- | --- | --- |
| Haplotype | rs2740210 | rs4813627 | Freq^a^ | Model Variables | Vocalizing^c^ |  | Instrumental Care^d^ |
| 1 | 1 | 1 | **.47** | H1 (Haplotype 1) | Ref |  | Ref |
| 2 | 1 | 2 | **.21** | H2 | -4.79 (-0.23) |  | 0.75 (0.30) |
| 3^e^ | 2 | 1 | .03 | H3 | - |  | - |
| 4 | 2 | 2 | **.29** | H4 | -60.1 (-3.10)** |  | 3.05 (1.31) |
|  |  |  |  | H2*Early care quality | - |  | 4.91 (1.62) |
|  |  |  |  | H4*Early care quality | - |  | 6.87 (2.35)* |

^a^ Frequency of haplotypes; bold values indicate major haplotypes; haplotype 1 was used as the base haplotype (reference group) in regression analyses. ^b^Values are unstandardized Betas (t-statistic). ^c^Model regressing maternal vocalizing onto haplotypes 2 and 4 controlling for the effects of parity, maternal age and education, infant gender and activity, and postpartum depression (CES-D score). ^d^Model regressing instrumental care duration onto haplotypes 2 and 4, controlling for the effects of parity, maternal age and education, infant gender and activity, and postpartum depression (CES-D score). ^e^Haplotype 3 was grouped with the intercept term due to low frequency. * p = 0.05; ** p = 0.01; *** p = 0.001.
