# Supplementary material for: Interaction between Oxytocin Genotypes and Early Experience Predicts Quality of Mothering and Postpartum Mood
Source: PLoS One. 2013 Apr 18;8(4):e61443. doi: 10.1371/journal.pone.0061443 (PMC3630168; doi:10.1371/journal.pone.0061443)
Supplement: Table S3 — Coefficient estimates of path models (using prenatal CES-D). (DOCX) [file pone.0061443.s004.docx]

Table S3. Coefficient estimates of path models (using prenatal CES-D)

|  | a_X_ | a_Z_ | a_XZ_ | R^2^ |  | b_X_ | b_M_ | b_Z_ | b_XZ_ | b_ZM_ | R^2^ |
| --- | --- | --- | --- | --- | --- | --- | --- | --- | --- | --- | --- |
| Coefficients | -4.22** | -0.87 | 2.48** | 0.05§ |  | -5.63** | 0.13 | 0.73 | 9.16** | 0.09 | 0.05* |
| CI 2.5% | -7.03 | -4.84 | 1.70 |  |  | -11.20 | -0.31 | -5.14 | 9.80 | -0.47 |  |
| CI 97.5% | -2.90 | 1.82 | 5.43 |  |  | -3.85 | 0.68 | 7.81 | 14.11 | 0.88 |  |

NOTE: N = 129-140 (values are based on non-imputed data). Coefficients a_1_, a_2_, and a_3_ were derived with formula 1 (see Methods) which uses *prenatal depression score* (CES-D) as the dependent variable and OXTrs2740210 genotype (grouped as genotype C/C versus genotype A/C and A/A) as the moderator (Z); coefficients b_1_ through b_5_ with formula 2, which uses *early care quality* as the predictor variable (X), *instrumental care* as the dependent variable, *prenatal depression score* as the mediator (M), and OXT rs2740210 genotype (C/C versus other) as the moderator (Z) (see Methods). CI: bias-corrected confidence intervals which are Bootstrap adjusted values (1000 bootstrap samples). §p=0.05, *p<0.05, ***p<0.01
