# Supplementary material for: Interaction between Oxytocin Genotypes and Early Experience Predicts Quality of Mothering and Postpartum Mood
Source: PLoS One. 2013 Apr 18;8(4):e61443. doi: 10.1371/journal.pone.0061443 (PMC3630168; doi:10.1371/journal.pone.0061443)
Supplement: Table S4 — Simple effects of path models (using prenatal prenatal depression score as a mediator). (DOCX) [file pone.0061443.s005.docx]

Table S4. Simple effects of path models (using prenatal prenatal depression score as a mediator)

|  |  | Stage | |  | Effect | | |
| --- | --- | --- | --- | --- | --- | --- | --- |
| rs2740210 Genotype |  | First | Second |  | Direct | Indirect | Total |
| C/C |  | -4.22** | 0.13 |  | -5.63** | -0.55 | -6.18** |
| A/C and A/A |  | -1.74 | 0.22 |  | 3.53 | -0.38 | 3.15 |
| Differences |  | 2.48** | 0.09 |  | 9.16** | 0.17 | 9.33** |

Note: N= 129-140. Simple effects computed using equation 25 in Edwards and Lambert (2007) using coefficients estimates from Table 5. Zs = 0 for C/C and 1 for A/C and A/A genotypes (rs2740210). Differences in simple effects were calculated by subtracting the A/C+A/A effects from the C/C simple effects. Significance values are based on bias-corrected boot-strap adjusted confidence intervals. **p<0.01.
